# Flooding and poverty: Two interrelated social problems impacting rural development in Tsholotsho district of Matabeleland North province in Zimbabwe

**DOI:** 10.4102/jamba.v10i1.455

**Published:** 2018-03-22

**Authors:** Ernest Dube, Oliver Mtapuri, Jephias Matunhu

**Affiliations:** 1 Department of Development Studies, Midlands State University, Zimbabwe; 2 School of Built Environment and Development Studies, University of KwaZulu-Natal, South Africa

## Abstract

Flooding and poverty are the two social problems that have coexisted within the rural communities of Tsholotsho district. As a result, both problems have negatively affected and disrupted the everyday pattern of lives of people living in the district. This study sought to highlight how the two problems combine to impact human societies. The objectives that the study sought to fulfil were to establish the impact of flooding on the development of rural communities, to analyse how poverty manifests itself in rural communities, to analyse the relationship that exists between flooding and poverty and to suggest ways for dealing with the two problems. A qualitative research approach, using interviews and observations, was used to gather data from the research participants. The study findings were that flooding impeded development through shifting of human populations, destruction of crops, shelter and livestock. Floods also affected human capital through causing injuries to members of the community. Poverty manifested itself in three ways – as a development barrier, a vulnerability amplifier and a non-discriminatory agent. The study further found that a strong relationship exists between flooding and poverty because of the fact that flooding causes or worsens poverty, whereas poverty increases flood vulnerability. The study concluded that the poor need government assistance to reconstruct shelter destroyed by floods. Furthermore, programs aimed at improving livelihoods of the poor are an indispensable imperative. This study informs policymakers and offers a methodological significance to development and disaster practitioners. It also adds to the body of literature on flooding and poverty.

## Introduction

Flooding and poverty are two social problems that have existed, and coexisted within rural communities. Whilst these two social evils have severely affected development programs in some rural communities, they have also manifested themselves into permanent features through lowering the standard of living in the communities. At times flooding has been found to exacerbate poverty levels and vice versa. However, community resilience and capacity to deal with both flooding and poverty have been found to be lacking in most human societies. In recent times, unprecedented incidents of flooding have resulted in serious disruption of human societies. For example, in the year 2010 alone, floods contributed 82.6% of all disasters that occurred on the African continent, a rise from 66.5% that was witnessed in 2009 (Balgah, Buchenrieder & Mbue 2015). Not to be outdone are the poverty levels that have bedevilled some communities, especially those in rural set-up. According to Matunhu (2012), Africa’s poverty continues to worsen as evidenced by the obvious low per capita income, low life expectancy, disease and hunger. As such, increased levels of poverty in Africa have forced people to live under vulnerable conditions. For instance, the United Nations Development Programme (UNDP 1992) stresses that poverty has forced people in some communities to live in temporary, unsafe shelter in crowded places, thereby exposing them to flood risk. Communities in Tsholotsho district, Matabeleland North province of Zimbabwe, have also been living with both flood risk and high levels of poverty for a long time now. Whilst flooding has been occurring seasonally in the district, poverty has been a daily feature. As it stands, these phenomena are likely to continue affecting people living in the district. Documented sources also suggest that the probability of an increase in incidents of flooding on the global stage in the future is very high (Wilby, Beven & Reynards 2008), whilst the situation of the poor also seems to be worsening at both the global and local stages (Mtapuri 2008). Hence, it is imperative to come up with measures for dealing with the problem of flooding, as well as measures towards eradicating poverty. The principal objectives of the study were to:

- establish impact of flooding on the development of rural communities
- analyse how poverty manifests itself in rural communities
- analyse the relationship between flooding and poverty
- suggest ways for dealing with the problems of flooding and poverty in rural communities.

## Statement of the problem

The setting of the problem(s) that this study confronts is in Tsholotsho district, Matabeleland North province in Zimbabwe. The district has been experiencing a spate of flooding events since the turn of the New Millennium. The floods have been a threat to development and humanity, affecting human lives, destroying property and damaging the environment. Whilst flooding has been impacting the communities, a high level of poverty has also been negatively affecting households in the district. Most people in the district are poor and lack basic necessities and needs that include decent shelter, food and clean water. However, flooding has worsened their poverty situation through affecting their livelihoods. Floods have damaged already unsuitable shelters, affected food stocks and also contaminated water sources. These two problems have therefore negatively affected development programs and initiatives in the district. Proper and meaningful measures are therefore needed in order to contain the impact of floods and eradicate poverty in the district. If nothing meaningful is done to avert the negative effects of flooding and high levels of poverty, people in Tsholotsho district would continue to suffer the lack of development because of the two problems of flooding and poverty.

## Review of related literature

Literature about the impact of flooding and poverty on development in rural communities is well documented. This section discusses the literature that is related to flooding and poverty, in line with the objectives of the study. The literature would help to broaden the understanding of flooding and poverty, either as independent agents or as a compound.

### The conceptual framework

Although flooding and poverty are two unwanted social problems with seemingly similar negative effects on rural development, they have, however, been conceptualised differently. Flooding occurs when water rises to submerge surrounding areas or inundate land that is normally dry (Kabubi 2011; Kates 1985; Stephen 2011). The sources of such water may be streams, dams, rivers and other basins located near human settlements. Consequently, flooding may result in the daily patterns of life of people living near the water sources being disrupted. It has been proved that ruptured dams or levees and the rapid melting of icebergs from the mountains can overwhelm rivers, leading to flooding of adjacent land or floodplains (Kabubi 2011). However, it is the adverse impact of flooding through claiming human life and destruction of property and livelihoods that communities are more worried about.

Just like flooding, poverty has been a source of worry to human communities as it has been found to impede rural development. Poverty has been conceptualised as consisting in any form of inequity, source of social exclusion and in living conditions essential to human dignity (Asselin 2002). Mack and Lansley (1985:39) define ‘poverty’ as ‘an enforced lack of socially perceived necessities’. Asselin (2002) further observes that the living conditions go hand in hand with the capacities of individuals, households and communities to fulfil their basic needs in the dimensions of nutrition, primary education, primary health care, sanitation, safe water, housing, income and community participation. The above definitions bring in the multidimensional concept of poverty, with Max-Neef (1992), as cited in Des Gasper (2007:475), writing about ‘poverties’ and not poverty. The multidimensionality of poverty means that poverty may be contextualised to some societies, for example, as lack of income, lack of education, lack of assets and to some extent, lack of technology. As such, poverty is also unwanted in human societies because it extremely affects development. As observed by the International Federation of Red Cross and Red Crescent Societies (IFRC 2000), extreme poverty can limit a community’s capacity to undertake development initiatives, as well as hinder implementation of risk reduction strategies. Dealing with poverty decisively within communities may ensure the smooth implementation of development projects and programs. It should be the responsibility of government to reduce poverty levels in societies, using funds collected through taxation and from aid (Matunhu 2012).

### How flooding can impact rural development

Flooding can affect and influence the pace of development in many rural areas. At times the progress of development in rural areas may be retarded because of the persistence of flooding in some places. Flooding can destroy property and infrastructure. According to Kabubi (2011), flood waters have awesome destructive power, such that structures that are poorly equipped to withstand the forces of water are subdued. For example, weak structures such as roads, bridges, houses and trees are usually affected. Even motor vehicles in flooded places can be swept away by strong flood waters. The destruction of property and infrastructure by floods reverses years of development gains, prompting a fresh start to carry out development programs.

Apart from destroying property and infrastructure, flooding may also pose health risks to members of societies in rural areas, thereby impacting on human capital. Communities affected by floods can be left without clean and safe drinking water, resulting in illnesses from outbreaks of deadly waterborne diseases (Campbell-Lendrum & Woodruff 2007). Human capital whose health is characterised by high levels of illnesses can hardly work towards the achievement of desired livelihood outcomes. Alson and Kent (2008) note that in rural Australia, repeated flooding impacted heavily on men’s health, resulting in high numbers of mental health cases and suicide rates. Gautam (2007) adds that the 2007 floods in Nepal affected many women’s health, resulting in anxiety, sleeplessness and feelings of helplessness. As such, development projects in rural communities may be stagnated or completed outside their timelines. Again, households affected by illnesses resulting from flooding are likely to be deficient in manual labour required for developmental purposes (Turnbull, Sterrett & Hilleboe 2013).

Floods have a more severe impact on women and children. The major reason being that women were created differently from men in terms of their physical and biological nature. Bulling (2011) observes that during floods, more women than men either suffer injuries or get killed. In rural areas, women are less likely to know how to swim. They can be restricted from swimming or running fast by their clothing; their roles as caretakers of children and older people, as well as cultural rules, restrict them from leaving their homes without the accompaniment of a male relative (Bulling 2011).

### Understanding poverty and its impact on rural development

Poverty eradication has been one of the major concerns of pro-development countries around the world because of its negative effects on societies. It is estimated that more than 700 million people globally are living below the $1.90 per day poverty line (World Bank 2015), making poverty a major threat to humanity. Although major strides have been taken at the global and local scales towards the elimination of poverty, the situation of the poor has been found to be worsening. Of note was the adoption of the Millennium Declaration in the year 2000 by countries signatory to the United Nations. The countries agreed to implement the Millennium Development Goals (MDGs), with MDG1 being focused towards eradicating poverty and extreme hunger. The main aim towards MDG1 was to half extreme poverty by the target date of 2015 (Mtapuri 2008). Just recently in 2015, at the World Conference in Sendai, states reiterated their commitment to building of resilience to disasters with a renewed sense of urgency within the context of sustainable development and poverty eradication (United Nations International Strategy for Disaster Reduction 2015). Despite these efforts, poverty levels have been increasing, especially in rural areas, owing to numerous factors including flood disasters.

### The link between flooding and poverty

Some major link exists between flooding and poverty, considering the manner in which both phenomena affect communities in a rural set-up. It is important to understand such a relationship so that communities are in a position to deal with both flooding and poverty. The UNDP (1998) acknowledges the direct connection between flood disaster and poverty, by stating that knowledge and understanding of poverty and socioeconomic characteristics of communities play a significant role in the management of disaster. The most obvious relationship is that both flooding and poverty act against development in communities. The two social problems can also complement each other in a negative manner. Whilst, on the one hand, flooding can worsen poverty levels, on the other hand, poverty too can exacerbate flood vulnerability and impact. This link between flooding and poverty is discussed in detail below.

#### Flooding leads to poverty and affects the poor most

By destroying property, dwellings, infrastructure, livelihoods and productive capital, flooding can leave some people in communities in a state of being poor. Those already poor can have their conditions worsened by a flood disaster, compared to those who are non-poor or wealthy. After the 2004 flooding in Bangladesh, the poor households impacted by the flood lost more than twice as much of their total income compared to the affected non-poor households (Brouwer et al. 2007). Bulling (2011) concurs and states that the lives and livelihoods of poor people living in flood plains, low lying coastal areas and steep slopes are in danger of flooding. This is contrary to the situation of the rich, who may live in the same dangerous areas and still survive flood impact because they have resources to build strong structures for shelter that are flood-resistant. The rich also can afford to replace their flood-damaged property because of their better financial positions and their ability to have flood insurance.

#### Flooding impacts heavily on the livelihoods of the poor

**FIGURE 1:**
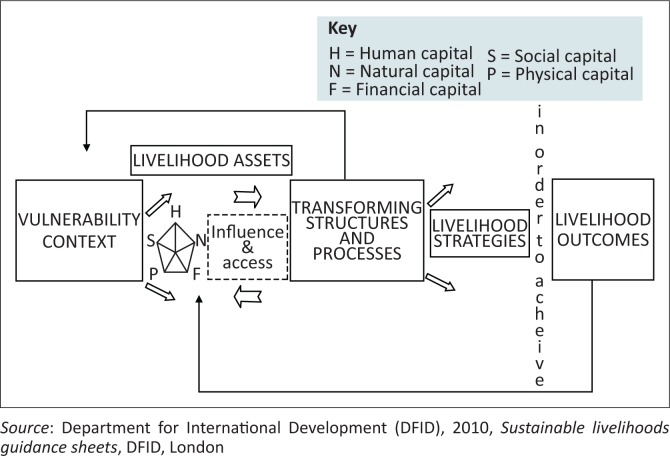
The sustainable livelihoods framework.

Flooding exacerbates poverty levels when it destroys livelihoods of the poor and affects their livelihoods capitals. Livelihoods of the poor that may be impacted on by flooding include crops, dams and boreholes, which may be affected resulting in people’s everyday pattern of life being disturbed. The livelihoods of the poor that are in danger to flood impact include their human, physical, natural, financial and social capitals or assets (Department for International Development [DFID] 2010), which form part of the sustainable livelihoods framework (SLF) (Figure 1).

Figure 1 is the DFID SLF which is an analysis to tool used to study and understand the livelihoods of poor communities. In the African context, the poor households or members of society are those who lack assets or income (Mtapuri 2011). The SLF is therefore used as a tool to provide the evidence base and to help ensure that proper interventions are tailor-made to have the positive impact (Allison & Horemans 2006; Tao & Wall 2009; Toner & Franks 2006). This framework has won the attention of policymakers and donors as it offers a fresh vision of a holistic and integrative focus with the capacity to analyse and comprehend the complexity of development in rural settings (Chambers & Conway 1992; Knutsson 2006). This study therefore found it relevant to adopt this approach as the study’s focus is on analysing and understanding the complexity of rural development in the poor communities of Tsholotsho affected by flooding. By impacting the poor’s livelihoods and livelihood assets or capitals (Figure 1), flooding can cause the capitals of the SLF to shrink, thereby perpetuating poverty within communities. For example, floods may cause human injury or death (human capital), destroy infrastructure (physical capital), cause land degradation (natural capital), affect business of financial institutions (financial capital) and disturb social networks (social capital). As a result, the capitals of the poor may shrink and poverty levels increase because affected communities may not be able to put them to maximum use in order to achieve positive outcomes. Flood impact is not only restricted to the livelihoods of the poor; rich households can also suffer the same impact. However, the rich always have a better way to minimise losses to their livelihoods and assets because they have means and resources to mitigate the flood impact.

Meaningful interventions are therefore required in order to improve the livelihoods of poor communities. The DFID’s SLF was developed in order to organise and improve organisations’ efforts to eliminate poverty in societies (ATHA 2014). In the context of this study, floods are part of the ‘Vulnerability Context’ shown in the framework (Figure 1).

#### Poverty contributes to flood vulnerability

Whilst flooding can make people poor, or worsen their situations, poverty has been seen as another factor contributing to flood vulnerability. This portrays flooding and poverty as two interrelated and interdependent social evils that combine to make human life more miserable. Their interrelatedness portrays flooding and poverty, functioning as a system to impact human societies. Decisively dealing with one problem or both would weaken the system. Therefore, poverty has been seen to contribute to flood vulnerability, as well as magnify flood vulnerabilities and flood impact. Sarmiento and Miller (2006) affirm that a population that lives in poverty is most significantly likely to be affected by flood hazards. However, poor households located uphill or in places not prone to flooding may suffer less flood impact compared to the poor living in flood prone areas.

**Poverty drives people to settle in hazardous places:** Some communities because of being poor are forced to settle in areas that are prone to flooding. The main reason for such manoeuvres being that the poor would be trying to improve their standards of living. Studies have also shown that people living in poverty are particularly vulnerable to floods, and such people are also often overrepresented in hazard prone areas (Winsemius et al. 2015). Poverty may therefore be the reason some people settle near rivers, and why those with low income have to seek housing in flood plains – areas previously avoided (Abramovitz 2001; Van Niekerk 2011). Loayza et al. (2012) add that in some rural areas, settling close to water offers cheaper transport opportunities for the poor and regular floods may improve their agricultural productivity. As such, it has become very difficult for the poor communities to avoid settling in areas where there is flood risk.

**Lack of decent shelter also worsens the poor’s flood vulnerability:** The need for human shelter by the poor has often been seen as another major contributor to their flood vulnerability. Poverty puts the poor in a position where they cannot fully perceive the presence and dangers posed by flood hazards. The poor may seek to build houses in places that are prone to flooding, thereby ‘constructing’ flood vulnerability. In the end, the poor have found themselves interacting with the flood hazard for considerably long periods. The poor people are also pushed to seek shelter in flood prone zones because of lower housing prices in those areas (Bin & Landry 2012; Husby et al. 2014). Their poverty situation diminishes their chances of being selective in the nature of dwellings to use, with some opting for cheap and substandard structures that cannot resist flood forces. When such structures are destroyed by floods not only do the poor lose the structures, but they also lose their property housed in the structures.

**Poverty also affects the poor’s capacity to respond to and recover from flood disasters:** Kundzewicz and Kaczmarek (2000) observe that apart from losing more to flooding, poor households also have a relatively lower capacity to deal with floods compared to households that are non-poor. One major reason being that the poor have less means to manage flood impact on their own, without relying on external assistance. They have lower access to savings, borrowing or social protection (Highfield, Peacock & Van Zandt 2014; Masozera, Bailey & Kerchner 2007). Minimising flood impact and eliminating poverty within societies is therefore a step in the right direction towards improving the standard of living and quality of life in rural communities.

## Research method and design

### Description of the study area

The study area is Tsholotsho district in Matabeleland North province in Zimbabwe. The district that is made up of 22 wards has an estimated population of 115 119 people (Zimstat 2012). The study was conducted in wards 5, 6 and 8, these being places with regular flooding. The places are also characterised with significant levels of poverty, with communities in the district highly dependent on subsistence farming to derive a living. High levels of flooding have been experienced in the district since the dawn of the New Millennium, worsening the situation of poverty-afflicted communities.

### Procedure

This study was carried out from August 2013 to September 2016. The aim of the study was to understand the relationship between flooding and poverty in affecting the poor in Tsholotsho district, through learning from their experiences. This study therefore used the qualitative approach and the interpretive research paradigm. The sampling was purposive and targeting a specific group of poor people with experience of flood impact. Data were gathered from 30 members of the community through interview and observation guides.

## Results and discussion

This section presents the results of the study and their discussion. The results are presented in response to the objectives that guided the study. In their discussion, the results are linked with the findings from previous studies by other researchers.

### Response to Objective 1: Impact of flooding on the development of rural communities

This study revealed that flooding impeded development in rural Tsholotsho through shifting of human populations, destroyed crops, shelter, livestock and resulted in human injuries. According to the respondents, some people impacted by floods were relocated on either a temporary or permanent basis. As a result of the relocation, some school-going children were heavily affected and missed lessons for weeks, as they waited for the floods to subside so that they could go to school. Respondents also narrated that those affected most were communities living close to Gwayi River, Bhudani and Gariya dams, and those settled in low lying areas. Because of flooding, some villagers along the Gwayi River had to relocate to safer areas across the River on a permanent basis. The areas are under Lupane district, meaning that the affected and relocated people lost their social networks and origins. According to respondents, their crops such as maize, sorghum, millet and groundnuts were destroyed by the floods, resulting in poor harvests between 2010 and 2015. Their shelter, most of which is made up of pole, mud and thatch, was also damaged, as narrated by the respondents from all the three wards. The consequences were that many families were left homeless and plunged into poverty. People’s livestock, especially chickens, goats and pets, were also affected. As indicated by the respondents, most of the small livestock and pets were washed away by floods during the night. These findings agree with a study on the Nigerian 1993 floods, where Adelye and Rustum (2011) found that flooding resulted in collapsed mud houses and washing away of livestock. The findings further confirm results of a previous study by Action Aid (2006), which concluded that flood is one of the major factors that prevent Africa’s population from escaping poverty level. In Tsholotsho district, respondents also indicated that some members of the community also suffered injuries when housing structures fell on them whilst they were sleeping. They were of the view that flood-risk areas should be identified, and flood hazard maps put in place, in order to improve people’s knowledge and increase awareness.

### Response to Objective 2: Manifestation of poverty in rural communities

Findings from the study showed that poverty manifested itself in three major ways in the rural communities of Tsholotsho district. Poverty manifested itself as a development barrier, a vulnerability amplifier and a non-discriminatory agent. As a development barrier, most households narrated that they were not able to make any meaningful development to improve their standard of living because of poverty. They indicated that they did not possess the necessary resources to drive development programs to benefit the community. For instance, lack of financial resources hindered the poor from purchasing suitable building materials for the construction of strong houses and infrastructure that can resist flood forces. As a result, the poor continued to live in substandard shelter without undertaking any meaningful development. This finding echoes results of a study by Winsemius et al. (2015), who found that poorer people have less financial resources to spend on housing, lack ability to pay for safety and are more likely to live in at-risk areas.

As a vulnerability amplifier, poverty forced some households to settle in areas prone to flooding, thereby increasing and perpetuating their vulnerability situation. These findings support a study by Adetunji and Oyeleye (2013) in Apete, Oyo State of Nigeria, who concluded that the location of the buildings on flood prone areas facilitated flooding. It was also observed that poor households in Butabubili area of Tsholotsho were using temporary shelter in the form of tents provided by humanitarian agencies, a scenario that worsened their vulnerability status, thereby underlining poverty as a vulnerability amplifier. Poverty was also found to be a non-discriminatory agent. From research observations, poverty was noted to be non-discriminatory in that it affected men and women, small and big size households, as well as old and newly established families.

### Response to Objective 3: Relationship between flooding and poverty

A strong relationship exists between flooding and poverty. Some respondents mentioned that because they were poor, they were forced to settle in flood prone areas in search of sustainable livelihoods. They stated that good farming prospects and availability of water along river banks and close to dams forced them to settle in those areas in anticipation of better harvest. This is in line with Bariweni, Tawari and Abowei (2012) who found that the higher the flood waters from the rivers, the greater the prospects for good harvests. According to respondents, areas along river banks have fertile soils, which they favoured because they cannot afford to buy fertilizer for their crops. Again, wild fruits and wild mushrooms found near the rivers supplemented their food reserves, as they had little income to buy food. As a result, their poverty status exposed them to flood hazards.

Apart from settling in flood prone areas, some respondents indicated that they had no financial and material resources to make them afford the construction of better and stronger housing. When the floods came, they were usually found wanting because their inferior structures were easily affected, resulting in the structures collapsing or being washed away. It was further established that the poor families already living with flood vulnerabilities were not willing to relocate because of the costs associated with relocation. The respondents indicated that they could not afford to relocate and start building new structures as they had no money or material resources. They suggested that relocation was only feasible if they were given assistance by the government and its cooperating partners, who in this case are non-governmental organisations and humanitarian agencies. Lutz, Sanderson and Scherbov (2008) also revealed that the poor in societies become victims of flooding because they end up living in flood prone areas.

Not only are the poor more vulnerable to flooding, but flooding also causes some members of communities to be poor. Members interviewed indicated that they were better off, in terms of property possessions, before they were affected by the floods. When the floods damaged their property and livelihoods, some households and individuals were left poor with nothing to call their own. This study therefore concluded that poverty increases flood vulnerability, and that flooding creates or worsens poverty levels. Both had negative effects on the communities in Tsholotsho district. Floods severely impact on community livelihoods and capitals, resulting in poverty or a rise in the existing levels of poverty. As a result, the poor communities living with flood vulnerability may depend on livelihoods and capitals that are severely diminished. Flooding therefore has a major bearing on poverty levels as livelihood capitals can shrink because of the flood impact.

## Recommendations

Having seriously considered its findings, the study recommends the establishment of human settlements away from flood prone areas. It is further recommended that the government should assist the poor in the construction of shelter through appropriate strong building materials. The government and stakeholders should also consider coming up with programmes aimed at improving livelihoods of the poor. Finally, the study recommends a multi-stakeholder approach that identifies flood prone areas, develops flood hazard maps and crafts appropriate, implementable flood management plans.

## Conclusion

The study concluded that flooding and poverty are related social problems that impede development planning and programming in rural communities. If these two problems are ignored by development planners and stakeholders, the study further concluded that no meaningful development can be achieved in many rural areas. Whilst flooding manifests itself as a destructive agent, poverty manifests itself differently. This study concluded that poverty manifests itself in three ways – as a development barrier, a vulnerability amplifier and a non-discriminatory agent. As a development barrier, poverty prevents members of the community from undertaking any meaningful development to improve their standard of living. As a vulnerability amplifier, poverty may force households to settle in areas prone to flooding, thereby contributing to their vulnerability situation. As a non-discriminatory agent, poverty may affect men and women, small and big size households, as well as old and newly established households. Furthermore, this study concluded that there exists a strong relationship between flooding and poverty, in that flooding may cause or worsen poverty, whilst poverty may increase flood vulnerability. Both are social problems with negative effects on human societies.
